# A Simplified Model of Adenine-Induced Chronic Kidney Disease Using SKH1 Mice

**DOI:** 10.3390/cells13242117

**Published:** 2024-12-20

**Authors:** Benjamin W. French, Joshua D. Breidenbach, Shereen G. Yassine, Bella Z. Khatib-Shahidi, Sara Kazmi, Caitlin M. Murphy, Humza S. Bashir, Evan M. Benson, Bivek Timalsina, Upasana Shrestha, Dhilhani Faleel, Satkeerth Boyapalli, Prabhatchandra Dube, Apurva Lad, Irum Syed, Deepak Malhotra, Amira Gohara, David J. Kennedy, Steven T. Haller

**Affiliations:** 1 Department of Medicine, College of Medicine and Life Sciences, University of Toledo, Toledo, OH 43614, USA; benjamin.french2@rockets.utoledo.edu (B.W.F.); joshua.breidenbach@rockets.utoledo.edu (J.D.B.); shereen.yassine@rockets.utoledo.edu (S.G.Y.); bella.khatibshahidi@rockets.utoledo.edu (B.Z.K.-S.); sara.kazmi@rockets.utoledo.edu (S.K.); caitlin.murphy@rockets.utoledo.edu (C.M.M.); humza.bashir@rockets.utoledo.edu (H.S.B.); evan.benson2@rockets.utoledo.edu (E.M.B.); bivek.timalsina@rockets.utoledo.edu (B.T.); upasana.shrestha@rockets.utoledo.edu (U.S.); fmohammedfaleel@mcw.edu (D.F.); satkeerth.boyapalli@rockets.utoledo.edu (S.B.); prabhatchandra.dube@utoledo.edu (P.D.); apurva.lad@utoledo.edu (A.L.); deepak.malhotra@utoledo.edu (D.M.); 2 Biochemistry and Biotechnology Group, Bioscience Division, Los Alamos National Laboratory, Los Alamos, NM 87545, USA; 3 Department of Bioengineering, College of Engineering, University of Toledo, Toledo, OH 43607, USA; 4 Department of Medical Microbiology and Immunology, College of Medicine and Life Sciences, University of Toledo, Toledo, OH 43614, USA; irum.syed@rockets.utoledo.edu; 5 Department of Pathology, College of Medicine and Life Sciences, University of Toledo, Toledo, OH 43614, USA; amira.gohara@utoledo.edu

**Keywords:** chronic kidney disease (CKD), adenine diet model, SKH1 elite mice, renal pathology, dermatological comorbidities

## Abstract

Commonly used adenine-induced chronic kidney disease (CKD) murine models often employ C57BL/6 mice; however, this strain has inherent limitations due to its natural resistance to developing key pathological features of CKD, such as tubulointerstitial fibrosis and inflammation. There have been attempts to overcome these barriers by using multiple concentrations of adenine-supplemented diets or by performing prolonged experiments up to 20 weeks. Here, we demonstrate that SKH1 Elite mice develop clinically relevant CKD phenotypes (e.g., polyuria, proteinuria, inflammation, and renal fibrosis) over the course of only 6 weeks of low-dose (0.15%) adenine supplementation. As a docile, immunocompetent, and hairless strain, SKH1 Elite mice offer several logistical advantages over C57BL/6 mice, including ease of handling and the ability to study dermal conditions, which are often secondary to CKD.

## 1. Introduction

Chronic kidney disease (CKD) is a gradual decrease in kidney function that affects ~13% of the global population—roughly 1 billion people [1]. Further, the last two decades have seen a rise in both the diagnosis of and deaths associated with CKD [2]. Great strides in understanding CKD causes and pathologies have come from utilizing several models in animals, including 5/6 bilateral nephrectomy and the more recent adenine-supplemented diet; however, these methods are limited. Kidney mass reduction by 5/6th bilateral nephrectomy is the most frequently used model of CKD in rodents. This model induces proteinuria and glomerular sclerosis and causes a persistent decrease in glomerular filtration rate (GFR) [3,4,5] via the removal of the upper and lower thirds of one kidney and the complete removal of the other. While 5/6th nephrectomy is a reliable means to simulate some CKD pathologies, it also incurs the risk of ureteral injury and bleeding, which contribute to a procedure mortality rate of up to 50% [6]. Moreover, variation in remnant kidney size can influence experimental outcomes.

The use of adenine to reliably produce a CKD phenotype in rodents is increasing, and its primary mechanism of injury has been investigated. Adenine is metabolized into 2,8-dihydroxyadenine, which then obstructs tubules by crystallization. This obstruction then initially leads to tubular damage and inflammation [6,7]. Recent models of adenine-induced CKD often employ C57BL/6 mice, which are known to be resistant to common CKD phenotypes [8], and many will use multiple concentrations of adenine diet (often starting at 0.2% adenine, followed by a lower adenine concentration in subsequent weeks) [6], prolonged studies (up to 20 weeks long) [9], or daily oral gavage [10]. While prolonged studies may accurately mimic the “chronic” aspect of CKD, study durations ≥ 20 weeks pose difficulties, including increased costs, higher risk of animal attrition due to age or disease progression, potential ethical concerns related to prolonged animal suffering, and logistical challenges in maintaining consistent experimental conditions over extended periods. These factors can complicate the interpretation of results and limit the feasibility of long-term studies. Other models, such as the 5/6th bilateral nephrectomy, are also commonly used but have high mortality rates (up to 50%) and induce their CKD phenotype in a short timeframe; many studies will conduct a 2/3rds nephrectomy first, followed by a total nephrectomy one week later. Studies of a more intermediate duration can model the chronic nature of CKD while offering a balance between experimental feasibility and ethical considerations. These studies allow for sufficient observation of disease progression and therapeutic effects without the extended costs while reducing ethical concerns and logistical challenges associated with longer studies. This makes studies with intermediate durations a practical alternative for investigating the pathophysiology of CKD and the long-term efficacy of potential therapeutics.

Patients with CKD, especially those with end-stage kidney disease (ESKD), often develop skin conditions secondary to renal disease [11]. Common observations in patients with ESKD include pallor, pruritus, xerosis, half-and-half nails, and cutaneous infections [11]. These are common features of dermatological conditions such as atopic dermatitis and psoriasis, which can increase mental and financial burdens; atopic dermatitis has one of the highest disease burdens among nonfatal diseases, as measured by disability-adjusted life-years [12]. The SKH1 mouse is one of the primary strains utilized in dermatological studies [13]. This immunocompetent, hairless strain is ideal for investigating skin-related conditions because their lack of fur facilitates easy observation and manipulation of the skin. Additionally, their intact immune systems enable more accurate study of immune-mediated skin diseases and their interactions with other systemic conditions. A CKD model in SKH1 mice would enable the study of CKD and the associated dermal comorbidities in a single, integrated model and would support the development of targeted therapies that address multiple facets of this disease. Herein, we investigated the suitability of an adenine diet-induced CKD model in SKH1 mice to concurrently examine nephrotic and dermal pathologies in the context of CKD.

## 2. Materials and Methods

### 2.1. Animals

Ten four-week-old male SKH1 Elite mice were purchased from Charles River (Wilmington, MA, USA) and acclimatized in the animal facility for 1 week before the study began. A group size of 5 was established using a power calculation; from our own previous work and from published studies, we anticipated a strong effect size (0.8), and we used an alpha value of 0.05 with a beta value of 0.8. Animals were kept under a 12/12 light/dark cycle at 20–25 °C for the entire study. Mice were given food and water ad libitum. To avoid recaging animals, mice were assigned to groups randomly by cage (n = 5/group). The control diet-fed mice started with an average body mass of 25.08 ± 1.47 g, while the adenine diet-fed mice had an average starting body mass of 24.76 ± 1.44 g (no statistical difference). Mice in both groups were housed in the same room, with identical environmental conditions. All animal studies were approved by the Institutional Animal Care and Use Committee at the University of Toledo (IACUC 108663).

Mouse bodyweight was tracked by massing each mouse three times per week. Animals who lost 20% of their starting bodyweight were assessed by the on-site veterinarian for overall health.

Utilizing published protocols for the use of adenine to induce a CKD phenotype in C57BL/6 mice, we tested multiple concentrations of adenine supplementation. Through this process, we discovered that SKH1 mice given the commonly used 0.2% adenine diet lost enough of their starting bodyweight within one week that the study should not be continued.

### 2.2. Adenine Diet

Low-protein, adenine-supplemented, and control diets were purchased from Envigo (Indianapolis, IN, USA). Both diets consisted of 2.5% protein from casein, 0.9% P, and 0.6% Ca. Both diets had identical formulations apart from adenine supplementation. The adenine-supplemented diet had a concentration of 0.15%.

In both the adenine-fed and control-fed mice, wet food was made freely available inside the cages, in addition to dry food in the overhead feeding rack. Wet food was replaced three times per week, at each point when animals were weighed. This consisted of 3–4 food pellets being placed in plastic Petri dishes and filled approximately halfway with water. One Petri dish in this fashion was used for every 2 mice in the cage. Mice in both groups received their respective diets for a total of 6 weeks.

### 2.3. Urine Collection

Twenty-four hours before the end of the study, mice were transferred to individual metabolic cages and provided with water and their associated diet ad libitum. Urine volumes were assessed at the end of the 24 h period by pipette.

### 2.4. Histology

Collected kidneys were cut longitudinally before being fixed in 10% formalin for 24 h and were then transferred to 70% EtOH. The fixed kidney sections were subsequently embedded in paraffin (FFPE), from which 5-micron sections were taken and placed on glass slides. Kidney slides were subject to hematoxylin and eosin (H&E) staining, and images were taken with a brightfield microscope with a 20× lens. A trained renal pathologist scored the kidney slides, blinded to sample and group IDs. The scores assessed total kidney section condition, inflammation, glomerular damage, interstitial damage, tubular atrophy, and calcification. Scores were assigned between 0 and 4, with 0 indicating no evidence of injury, 1 as mild presentation, 2 as moderate, 3 as severe, and 4 as extensive.

### 2.5. Measures of Proteinuria

Urine samples from both groups (n=5) were diluted 1:200 in UltraPure sterile water before being used in the following assays. Six standards were used for both assays, using concentrations of 0 mg/mL (UltraPure water), 0.05 mg/mL, 0.1 mg/mL, 0.3 mg/mL, 0.5 mg/mL, and 1 mg/mL of bovine serum albumin (BSA).

#### 2.5.1. Bradford Assay

The Bradford assay reagent was purchased from Bio Rad (Cat #5000002; Hercules, CA, USA) and was used according to the manufacturer’s directions. Briefly, 5 µL of standard or sample was added to a well in a 96-well plate, followed by 250 µL per well of the Bradford dye/reagent before mixing via plate shaker for 15 s. The plate was incubated at room temperature for 5 min. The absorbance was then measured at 595 nm in a plate reader. Standards and samples were performed in triplicate.

#### 2.5.2. Detergent Compatible Lowry Assay

The Bio Rad Detergent Compatible (DC) Lowry assay (Cat #5000112; Hercules, CA, USA) was used alongside the Bradford assay as described above and using the same set of standards. The DC protein assay was conducted per the manufacturer’s directions. Briefly, 5 µL of sample or standard was added to each well in a 96-well plate, followed by 25 µL of reagent A and 200 µL of reagent B. The 96-well plate was then briefly mixed on an orbital shaker for ~15 s before incubation at room temperature in the dark for 15 min. The absorbance was then measured at 750 nm.

The differential measurement of urinary proteins and peptides using the Bradford and Lowry assays offers a novel approach to assessing renal injury. The Bradford assay primarily detects intact proteins, while the DC Lowry assay measures both proteins and peptides. By subtracting the Bradford assay results from those of the Lowry assay, the peptide content in urine can be estimated. This method has been utilized to evaluate renal function and detect early signs of renal pathology [14,15].

### 2.6. Measurement of Cystatin C

Cystatin C is a robust marker of renal damage, as a low molecular weight protein freely filtered by the glomeruli. Further, cystatin C is less affected by variability in diet and muscle mass than some other renal markers, making it a reliable means for assessing renal damage [16,17,18]. Levels of cystatin C were measured from plasma collected from mice on both diets. Blood and plasma were separated immediately after collection by centrifugation at 2000× *g* for 10 min in a centrifuge pre-cooled to 4 °C. Plasma was carefully pipetted off and stored in −80 °C until use.

Cystatin C was measured using the Mouse Cystatin C ELISA Kit (Cat #ab201280) from Abcam (Waltham, MA, USA) following the manufacturer’s protocol. All samples, standards, and reagents were allowed to equilibrate to room temperature before starting the assay. Briefly, 8 standards of cystatin C were made at concentrations of 15,000 pg/mL; 1500 pg/mL; 750 pg/mL; 375 pg/mL; 188 pg/mL; 93.8 pg/mL; 46.9 pg/mL; 23.4 pg/mL; and 0 pg/mL. All standards and samples were run in duplicate, and the average of both samples was used for analysis. Fifty microliters of either standard or sample was added to each well, along with 50 µL of Abcam’s Antibody Cocktail. The plate was sealed and incubated at room temperature on a plate shaker set to 400 rpm. Each well was then washed 3× with Wash Buffer PT. One hundred microliters of TMB Development Solution was then added to each well and allowed to incubate for 10 min in the dark on a shaker set to 400 rpm. After the 10 min, 100 µL of Stop Solution was added to each well, shaken gently but thoroughly, and the absorbance was then measured at 450 nm.

### 2.7. RNA Isolation and Reverse Transcription Polymerase Chain Reaction

Extraction of RNA from kidney tissue and subsequent complementary DNA (cDNA) synthesis and RT-PCR was conducted using Qiagen’s (Germantown, MD, USA) workflow system. Briefly, 20–30 mg of frozen kidney tissue was massed and subject to RNA isolation in the QIAcube HT with a QIAzol/chloroform extraction solution. RNA quantity and purity were measured by ND1000 (Thermo Scientific, Waltham, MA, USA). All 260/280 values were ≥1.9, and all 260/230 values were ≥1.8. Approximately 500 ng of extracted RNA was used for the preparation of cDNA with the RT2 First Strand Kit (QIAGEN, Cat. #330404). RT-PCR was performed in QIAGEN’s Rotor-Gene Q thermocycler, calculating gene expression using relative change in cycle threshold value as we have described (∆Ct) (Khalaf et al., 2022). Data obtained from renal RT-PCR is presented in Log_2_ fold change (Log_2_FC).

RNA extraction and preparation of cDNA from heart tissue was performed in the same fashion. To eliminate interindividual variability in the array analysis, all cDNA from the same groups were pooled within their group, and each pooled sample was run in triplicate in the RT2 Profiler PCR Array for Mouse Cardiovascular Disease (Q-100 format, Qiagen, Germantown, MD, USA, Cat. No.: 330231, GeneGlobe ID: PAMM-174Z). Array results were analyzed through the Qiagen GeneGlobe PCR Array Analyzer. Cardiovascular disease array results were considered significant for *p*-values <0.05 and a fold change ≥1.5.

### 2.8. Statistics

Statistics were conducted using GraphPad Prism. All data were subjected to the Shapiro–Wilk normality test to assess normal distribution. Unpaired *t*-tests were applied to gene expression data to determine significance. For tracking weight over time, multiple *t*-tests were conducted at each time point. For RT^2^ PCR Array analysis, the GeneGlobe Analysis website was utilized, and results were considered significant for *p*-values ≤ 0.05 and a fold change ≥1.5 (https://geneglobe.qiagen.com/us/analyze, accessed 26 September 2024). *p*-values are reported as *, **, ***, and **** correlating to values of *p* ≤ 0.05, ≤0.01, ≤0.001, and ≤0.0001, respectively.

## 3. Results

### 3.1. Mouse Bodyweight Reduced by Adenine Diet

**Figure 1:**
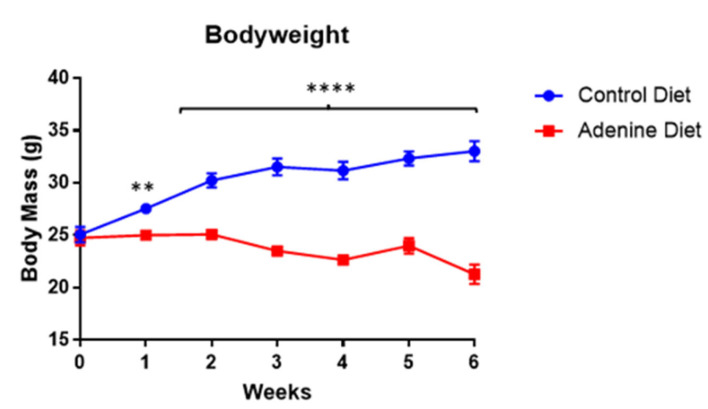
Bodyweight of SKH1 Elite male mice over the course of the 6-week study (n = 5/group). Weights displayed are the average within the group with standard error from the mean. Week 0 is the starting bodyweight, and each mass thereafter is at the end of the week displayed (week 1 = day 7 of the study). Significance is indicated on the figure where ** and **** correspond to *p*-values ≤ 0.01 and 0.0001, respectively. Statistics by individual *t*-tests at each time point.

Bodyweight in both the control diet-fed and adenine diet-fed mice was tracked throughout the course of the 6-week study. The control diet-fed mice consistently and incrementally gained weight over the course of the study, with an average of 7.96 ± 1.50 g increase in bodyweight. On the other hand, the adenine-fed mice maintained or lost weight over the course of the study, losing an average of 3.48 ± 2.41 g from their starting weight (Figure 1).

### 3.2. Adenine Diet Induces Renal Histopathological Damage

**Figure 2:**
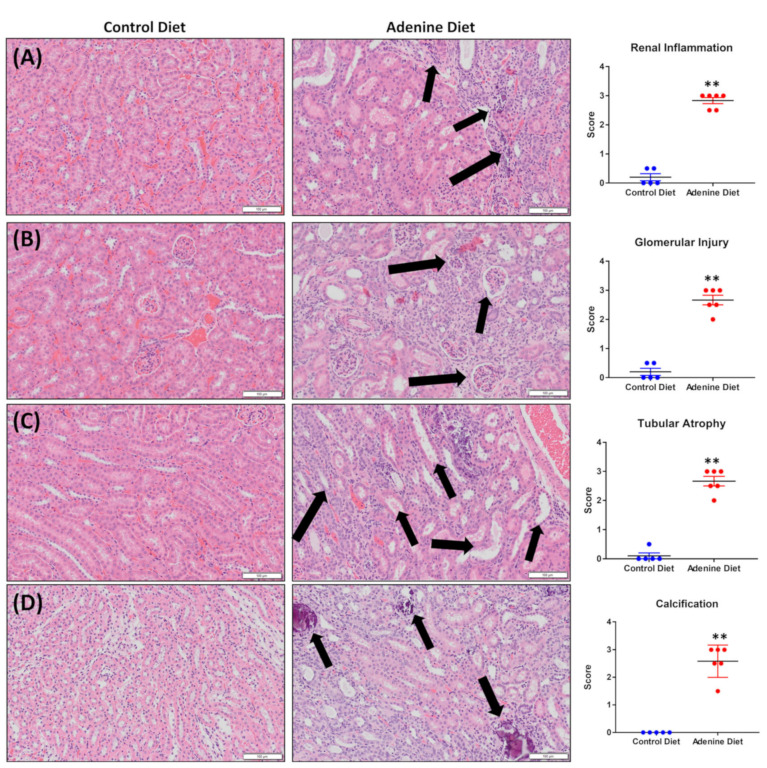
Hematoxylin and eosin staining of fixed kidney sections. All representative images appear at 10× magnification. The images provide histological evidence of adenine-driven (**A**) renal inflammation, where black arrows indicate infiltrating immune cells; (**B**) glomerular damage, where black arrows indicate damaged glomeruli; (**C**) tubular atrophy, where black arrows indicate atrophied tubules; and (**D**) calcification, where black arrows indicate calcified regions of tissue. The graphs display the results of blind scoring of tissue inflammation, glomerular injury, tubular atrophy, and calcification by a trained renal pathologist, where blue points are data from control diet-fed mice and red points are data from adenine diet-fed mice. Statistics for histology scoring were conducted using the Mann–Whitney test. Significance is indicated on the figure where ** corresponds to a *p*-value ≤ 0.01.

Renal histological analysis showed evidence of histopathological damage induced by the adenine diet against the control. Initial observations included elevated renal inflammation (Figure 2A, *p* = 0.0022), glomerular damage (Figure 2B, *p* = 0.0043), tubular atrophy (Figure 2C, *p* = 0.0043), and calcification (Figure 2D, *p* = 0.0043). Blind scoring of these features revealed significantly more renal damage in the adenine-fed mice compared to the control mice across all categories listed.

### 3.3. Adenine Diet Induces Multiple Markers of Renal Injury

**Figure 3:**
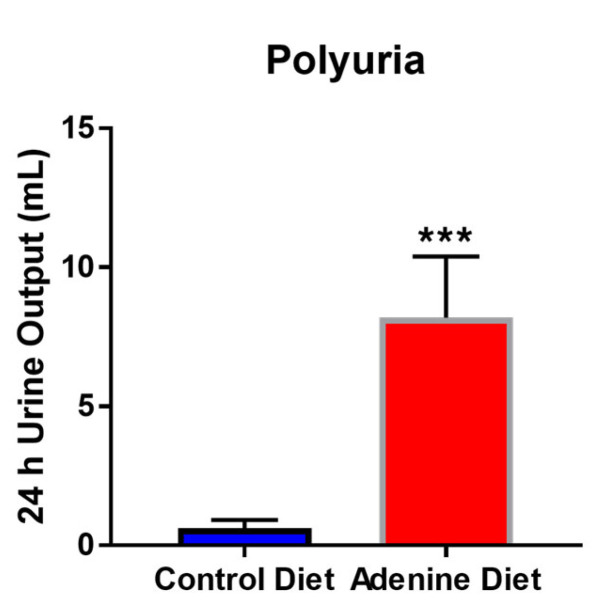
Total urine output over 24 h in metabolic cages from control diet-fed mice and adenine diet-fed mice (n = 5/group). Significance assessed by unpaired *t*-test, where *** indicates a *p*-value < 0.001.

**Figure 4:**
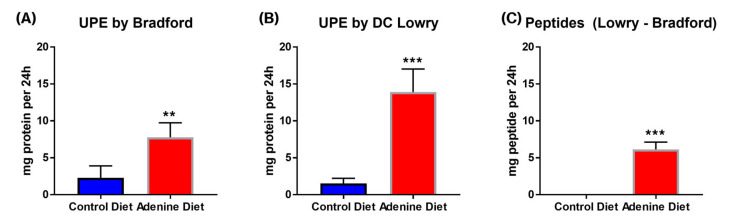
24 h urinary protein excretion (UPE) assessed by (**A**) the Bradford protein assay calculated as total protein, (**B**) the DC Lowry assay, and (**C**) peptides, as calculated by subtracting full-length proteins (Bradford) from the protein + peptide fragments (Lowry) (n = 5/group). Significance assessed by unpaired *t*-test, where ** and *** indicate a *p*-value < 0.01 and <0.001, respectively.

Total 24 h urine output in the adenine-fed mice was significantly higher than that of the control diet-fed group. The control diet-fed mice excreted an average of 0.6125 ± 0.270 mL of urine over the 24 h period, while the adenine diet-fed mice excreted an average of 8.2 ± 1.97 mL of urine within the same timeframe; the adenine-fed mice urinated an average of 13× the volume than that of the control mice (Figure 3, *p* = 0.0003). Proteinuria measured by both the Bradford (for full-length protein) and DC Lowry Protein (for protein and peptide fragments) assays shows elevated 24 h protein/peptide secretion in the adenine diet-fed mice compared to the control group. The Bradford assay showed that the adenine-fed mice had an average of 7.77 ± 1.76 mg protein/24 h compared to the control 2.27 ± 1.41 mg protein/24 h; similarly, the DC protein assay showed adenine-fed mice had an average of 13.9 ± 2.78 mg protein/24 h against the 1.52 ± 0.61 mg protein/24 h from the control mice (Figure 4A) Bradford *p*-value = 0.0029, B) DC *p*-value = 0.0001). Similarly, when calculating the 24 h urinary peptide excretion, we noted that while there were negligible levels in the control group, there was significant excretion in the adenine diet-fed group, indicating tubular damage. The adenine diet-fed mice lost an average of 6.13 ± 1.99 mg peptide/24 h, compared to the control group’s average of −0.76 ± 0.86 mg peptide/24 h.

**Figure 5:**
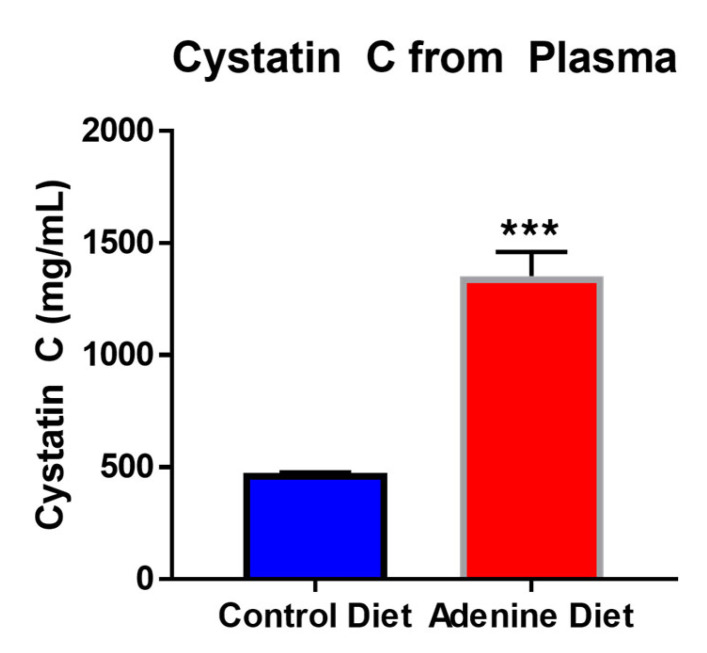
Levels of circulating cystatin C from control diet-fed mice and adenine diet-fed mice (n = 5/group). Significance was determined by an unpaired *t*-test and is indicated on the figure where *** corresponds to a *p*-value ≤ 0.001.

Circulating (plasma) cystatin C was measured by ELISA at the endpoint of the study. The adenine-fed mice had an average circulating cystatin C level that was 2.8× higher than that of the control diet-fed mice: 1352.5 ± 284.9 pg/mL vs. 473.7 ± 6.53 pg/mL, respectively (Figure 5).

**Figure 6:**
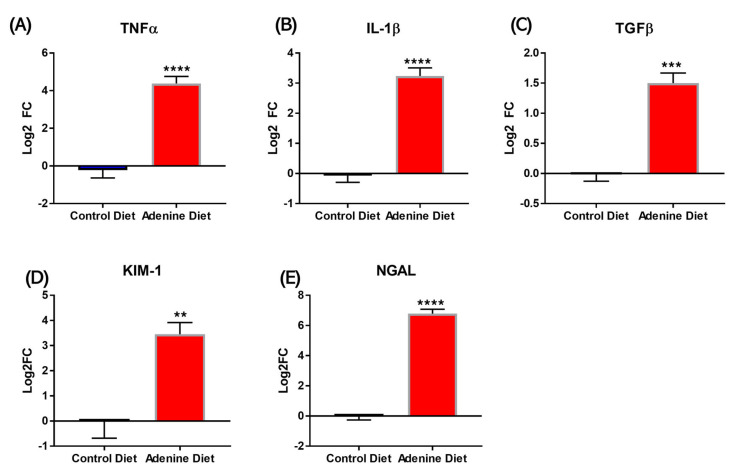
RT-PCR analysis of control diet-fed mouse kidneys and adenine-diet-fed mouse kidneys (n = 5/group). The figures represent the Log_2_ (Fold Change) in the expression of (**A**) TNFα; (**B**) IL-1β; (**C**) TGFβ; (**D**) KIM-1; and (**E**) NGAL. Significance assessed by unpaired *t*-test of adenine diet against control diet, where **, ***, and **** represent *p*-values < 0.01, <0.001, and <0.0001, respectively.

Kidney tissue from adenine-fed mice demonstrated significantly higher levels of tumor necrosis factor alpha expression (TNFα Log_2_FC = 4.38 ± 0.065; Figure 6A, *p* < 0.0001), interleukin-1 beta (IL-1β Log_2_FC = 3.24 ± 0.53; Figure 6B, *p* < 0.0001), transforming growth factor beta (TGFβ Log_2_FC = 1.50 ± 0.29; Figure 6C, *p* = 0.0001), Kidney Injury Marker 1 (KIM1 Log_2_FC = 3.45 ± 0.93; Figure 6D, *p* = 0.0031), and neutrophil gelatinase-associated lipocalin (NGAL Log_2_FC = 6.79 ± 0.51; Figure 6E, *p* < 0.0001) relative to the control-diet mice.

### 3.4. Cardiovascular Gene Expression Reveals Adenine-Induced Cardiac Damage

Analysis of pooled cDNA from control versus adenine-fed heart tissue revealed multiple markers of cardiovascular disease were significantly modified by the 6-week diet. We detected 35 differentially expressed genes in the adenine-fed heart tissue, including genes that regulate cell growth, cardiac remodeling, cellular signaling, and structural proteins (Table 1 and Table S1). Of note, Collagen 1a1 (fold change 2.54; *p* = 0.001451), 3a1 (fold change 3.11; *p* = 0.01283), and 11a1 (fold change 23.73; *p* = 0.004893) were all significantly upregulated in the adenine-fed group, indicative of cardiac fibrosis and remodeling. Other affected cardiovascular remodeling genes include fibronectin 1 (Fn1; fold change 3.91; *p* = 0.002427) and coagulation factor II receptor (F2r, which encodes for proteinase-activated receptor 1, fold change 1.94; *p* = 0.031817). Additionally, genes that regulate cellular transporters, signaling, and immune response were also significantly upregulated, including C–C motif chemokine ligand 2 (Ccl2 fold change 2.21; *p* = 0.003723), kelch-like family member 3 (Klhl3 fold change 1.94; *p* = 0.036163), and mitogen-activated protein kinase 1 (Mapk1 fold change 1.2; *p* = 0.006052). Fold changes and *p*-values for all genes tested on the RT^2^ Profiler Array are listed in the (Supplemental Table S1).

## 4. Discussion

Here, we provide multiple lines of evidence that support the use of a single concentration of adenine in a low-protein diet over the course of 6 weeks in the SKH1 mouse in order to model CKD. Previous models of adenine-induced CKD typically involved the use of multiple diet formulations applied at several time points, higher concentrations of adenine, or longer durations on the diet [6,9,10]. Moreover, previous studies often use C57BL/6 mice, which are resistant to many of the clinical pathologies seen in patients with CKD [19]. The 5/6th bilateral nephrectomy model, while very popular, has its own shortcomings. This model has a high mortality (up to 50%) and induces its CKD phenotype within one week. These factors raise the cost of research and suppress therapeutic innovation. While the model herein may be somewhat limited by its intermediate duration to develop key features of CKD (6 weeks), it nevertheless shows promise as a method of studying CKD due to the strength of the phenotype, its chronic development, and simplified application.

Chronic kidney disease is a multifaceted condition characterized by progressive renal dysfunction, often accompanied by systemic complications such as weight loss, proteinuria, and tubular injury, which collectively reflect the interplay between glomerular and tubular damage [20,21]. Many patients experience damage to both glomerular and tubulointerstitial structures, with tubular damage often being a major contributor to disease development and progression [22,23,24,25]. In the current study, adenine-fed mice demonstrated weight loss after the first week on the adenine-supplemented diet (approximately a 34% reduction). The most similar study of which we are aware utilized C57BL/6 mice on a 0.2% adenine diet for 6 weeks and saw an almost identical reduction in bodyweight, but this model continued for an additional 4 weeks past this point and focused on changes to bone structure rather than cystatin C, polyuria, proteinuria, or gene expression in either the kidneys or heart [8]. Similarly, another study used C57BL/6 mice (starting at 8 weeks of age) and applied a 0.15% adenine diet for 20 weeks; this study showed a CKD phenotype but did not demonstrate weight loss [9].

Histological scoring showed an increase in inflammation, glomerular injury, tubular atrophy, and calcification, with each category having an average score 2 or more higher in the adenine-fed group compared to control (scale 0 to 4). Other important clinical observations in CKD patients are an increase in urine volume (polyuria) and protein content within the urine (proteinuria). The 0.15% adenine diet reliably induced both of these outcomes, with the adenine-fed mice urinating out 13× the volume within the same 24 h period (average of 8.2 mL/24 h in the adenine-fed mice versus an average of 0.6125 mL/24 h), 3.4× the protein secretion by the Bradford assay (average of 7.77 mg/24 h in the adenine-fed mice versus 2.27 mg/24 h), and 9.1× the protein secretion by the DC Lowry assay (average of 13.9 mg/24 h in the adenine-fed mice versus 1.52 mg/24 h).

The observed increases in both full-length proteins and peptides in the urine provide valuable insights into the mechanisms of renal injury. Other researchers have identified the primary mechanism by which adenine induces CKD, namely, the crystallization of metabolized adenine (2,8-dihydroxyadenine), creating obstructions within renal tubules [7]. Our model follows this pattern, with strong tubular damage as evidenced by both histopathology and elevated levels of urinary peptides. In addition, we also noted significant glomerular damage as well, indicated both histologically as well as by elevated levels of intact full-length protein in the urine (Bradford results). Normally, the glomerular filtration barrier prevents the passage of larger proteins such as albumin into the urine. The increased excretion of full-length proteins in our model aligns with findings in other studies, where glomerular injury or permeability defects are a hallmark of proteinuria in renal disease. The presence of increased peptides alongside full-length proteins (DC Lowry assay subtraction) suggests additional tubular dysfunction. Renal tubules normally reabsorb and degrade filtered proteins into peptides or amino acids for reuse. The elevated peptide levels in our study, derived by subtracting Bradford assay results (full-length proteins) from Lowry assay results (proteins + peptides), point to impaired tubular reabsorption and/or increased degradation of filtered proteins, consistent with tubular injury. The fact that we noted an increase in both full-length proteins and peptides suggests that both the glomeruli and tubules are compromised in the adenine diet model. This pattern of injury is supported by the histological findings and is consistent with several studies [14,15], where increased full-length proteins correlate with glomerular filtration barrier damage, while elevated peptides reflect tubular overload or a diminished ability to handle filtered proteins effectively. Further, this mimics the patterns of damage often seen in patients with CKD [22,23,24,25].

Markers of renal damage and inflammation were also increased by dietary adenine supplementation. These markers showed strong changes, where TNFα (Log_2_FC = 4.38), IL-1b (Log_2_FC = 3.24), TGFb (Log_2_FC =1.50), KIM-1 (Log_2_FC = 3.45), and NGAL (Log_2_FC = 6.79) in the adenine diet group versus control. While there are many markers of CKD, these are strong indicators of renal inflammation and fibrosis in both human patients and CKD models [22,26,27,28,29].

In this study, cystatin C was used as a marker for renal damage as it appears to be a more sensitive and accurate marker than creatinine. Cystatin C is a low molecular weight protein that is filtered by the glomeruli and metabolized in renal tubules. Cystatin C is less affected by muscle mass and diet compared to creatinine, as several previous studies have indicated [16,17,18]. In the current study, we found a significant increase in circulating cystatin C (a 2.86× increase), providing additional support for the use of adenine to induce a CKD phenotype in SKH1 mice. The elevated circulating cystatin C, taken in tandem with our histological, biochemical, and genetic evidence, indicates both glomerular and tubular injury from dietary adenine supplementation.

Another key phenotypic feature of CKD is the development of cardiovascular complications, which are tightly linked to both the progression of renal dysfunction and the systemic inflammatory state induced by CKD. In our adenine diet-induced CKD model, we observed evidence of early cardiovascular remodeling, including significant upregulation of key genes associated with cardiac fibrosis and inflammation, such as Collagen 1a1, Collagen 3a1, and Collagen 11a1, as well as markers of immune activation like Ccl2 and TGFβ. These findings align with the well-documented cardio-renal syndrome, where kidney injury exacerbates cardiac dysfunction through overlapping pathways of inflammation, oxidative stress, and vascular remodeling. Collagens are especially important in the progression of cardiac fibrosis; all three collagens measured in this assay (Col1a1, 3a1, and 11a1) were significantly upregulated, signaling the initiation of cardiac fibrosis alongside the adenine-induced CKD. With 29 cardiovascular disease-related genes differentially regulated but limited histopathological evidence of cardiac fibrosis, this model demonstrates a strong kidney phenotype and the early development of secondary heart damage. Existing models demonstrate cardio-renal syndrome by using higher concentrations of adenine, implementing the diet for longer (up to 20 weeks), and using rats [9,30]. For example, one study applied a 0.15% adenine diet to C57BL/6 mice for 20 consecutive weeks and presented a CKD phenotype alongside reduced cardiac ejection fraction, fractional area change, and left ventricular end diastolic volume [9].

This model of CKD is uniquely advantageous in that it will enable easier and more direct study of skin conditions that arise secondary to CKD in an immune-competent system. Patients with CKD often develop dermatological conditions as a result of CKD [31,32]. The risk for these conditions increases as CKD progresses and is most commonly found in patients on dialysis [31]. SKH1 mice are a common strain used in dermatology studies [13]. By adapting the adenine-induced CKD model into the SKH1 mouse, researchers are equipped with better tools for complicated disease interactions. CKD is just one of several related diseases that affect both the kidneys and skin; other examples include diabetes [33], obesity [34], and cardiovascular disease [35].

Our CKD model is efficiently implemented using the SKH1 mouse strain, requiring only a single concentration of an adenine-rich diet administered over a 6-week period. The SKH1 mimics clinically relevant CKD pathologies, including polyuria, proteinuria, weight loss, elevated renal inflammation, and histopathological changes in the kidney (e.g., calcification, tubular atrophy, and glomerular damage), as well as multiple markers of cardiac injury. Importantly, this model exhibits evidence of mixed tubular and glomerular involvement, a common occurrence in human patients. Many etiologies of CKD, such as diabetic nephropathy or AKI, often cause damage to both structures, using the breakdown of tubular function as a primary mechanism of injury [24]. Additionally, the SKH1 mouse serves as a valuable model for investigating skin conditions that are secondary to CKD, offering a unique model to examine the broader impacts of CKD on dermatological health.

## Figures and Tables

**Table 1:** Cardiac remodeling, stress and immune response, transporter, and signaling genes significantly altered by the 6-week course of a 0.15% adenine diet compared to control. Fold change is relative to control animals (n = 5/group). For a complete list of altered genes, see Supplemental Table S1.

Gene Symbol	Fold Change	*p*-Value	Categorization
Col1a1	2.54	0.001451	Cardiac Remodeling
Col3a1	3.11	0.01283	
Col11a1	27.73	0.004893	
Fn1	3.91	0.002427	
F2r	1.94	0.031817	
Ccl2	2.21	0.003723	Stress and Immune Response
Klhl3	1.94	0.036163	Transporters
Ar	2.13	0.001831	Signal Transduction

## Data Availability

Data are contained within the article or Supplementary Material. Further inquiries may be directed at the corresponding authors.

## Supplementary Materials

The following supporting information can be downloaded at https://www.mdpi.com/article/10.3390/cells13242117/s1. Supplemental Table S1 contains the *p*-values and fold changes for all genes assessed in the Mouse Cardiovascular Disease (Q-100 format, Qiagen, Germantown, MD, USA, Cat. No.: 330231, GeneGlobe ID: PAMM-174Z) RT^2^ PCR Array. Bolded items indicate a *p*-value < 0.05.
