# Supplementary material for: A Simplified Model of Adenine-Induced Chronic Kidney Disease Using SKH1 Mice
Source: Cells. 2024 Dec 20;13(24):2117. doi: 10.3390/cells13242117 (PMC11726765; doi:10.3390/cells13242117)
Supplement: Supplementary file 1 [file cells-13-02117-s001.zip › cells-3364123-supplementary.pdf]

Supplementary Materials:

Position	Symbol	Fold Change	p-value
A01	Ace	1.44	0.200812
A02	Actc1	0.78	0.287466
**A03**	**Adra1a**	**1.60**	**0.045969**
**A04**	**Adra1b**	**1.78**	**0.007651**
A05	Adra1d	2.57	0.207731
A06	Adrb1	1.10	0.615558
A07	Adrb2	1.75	0.160937
A08	Adrb3	0.18	0.056396
**A09**	**Aebp1**	**3.86**	**0.016284**
A10	Agtr1a	1.13	0.123142
A11	Anxa4	1.47	0.073937
**A12**	**Ar**	**2.13**	**0.001831**
B01	Atp2a2	0.70	0.255033
B02	Atp5a1	0.97	0.749668
**B03**	**C6**	**14.71**	**0.002727**
B04	Ccl11	1.46	0.058392
**B05**	**Ccl2**	**2.21**	**0.003723**
**B06**	**Ccnd1**	**1.49**	**0.006083**
B07	Cdkn1b	0.81	0.222410
**B08**	**Col11a1**	**23.73**	**0.004893**
**B09**	**Col1a1**	**2.54**	**0.001451**
**B10**	**Col3a1**	**3.11**	**0.012830**
B11	Creb5	1.36	0.149760
B12	Crem	1.44	0.221093
**C01**	**Cryab**	**0.38**	**0.004804**
**C02**	**Crym**	**3.97**	**0.002480**
**C03**	**Ccn2**	**2.69**	**0.002003**
**C04**	**Cxcl12**	**1.32**	**0.000221**
C05	Dcn	0.91	0.464067
C06	Dmd	0.93	0.663145
C07	Dusp6	1.70	0.089440
C08	Enah	0.81	0.153766
C09	Epor	0.86	0.498077
**C10**	**F2r**	**1.94**	**0.031817**
**C11**	**Fn1**	**3.91**	**0.002427**
**C12**	**Frzb**	**2.48**	**0.039157**
D01	G0s2	0.66	0.018168

D02	Gja1	0.66	0.014853
D03	Hmgcl	1.19	0.324507
D04	Hmgcr	1.28	0.155843
D05	Hmgn2	1.16	0.238915
**D06**	**Klhl3**	**1.94**	**0.036163**
**D07**	**Maoa**	**2.54**	**0.004786**
D08	Map2k5	1.78	0.286042
**D09**	**Mapk1**	**1.20**	**0.006052**
D10	Mapk8	1.01	0.901781
D11	Mmp13	1.24	0.652148
D12	Msi2	0.89	0.110379
**E01**	**Myh10**	**2.16**	**0.035898**
E02	Myh6	0.90	0.408269
E03	Ndufb5	0.51	0.025393
E04	Nebl	0.91	0.463710
E05	Nfia	1.21	0.243345
E06	Nkx2-5	3.37	0.055550
E07	Nppa	1.36	0.067185
E08	Nppb	1.13	0.187491
E09	Npr1	1.27	0.122600
E10	Npr2	0.81	0.153852
**E11**	**Npr3**	**1.70**	**0.018411**
**E12**	**Nr3c1**	**1.22**	**0.017828**
F01	Nr3c2	1.59	0.109305
F02	Pde3a	0.70	0.018968
F03	Pde3b	1.00	0.892025
F04	Pde5a	1.42	0.071144
F05	Pde7a	1.19	0.259446
**F06**	**Postn**	**1.36**	**0.019613**
**F07**	**Ptn**	**1.90**	**0.024874**
F08	Rarres1	0.77	0.388159
**F09**	**Rassf1**	**1.35**	**0.000593**
F10	Ren1	1.69	0.622013
F11	Rtn4	0.79	0.023424
F12	S100a1	0.88	0.311747
G01	S100a8	1.21	0.234412
**G02**	**Sfrp4**	**8.01**	**0.014418**
G03	Slc12a1	0.58	0.151196
G04	Snca	0.97	0.556951
G05	Spock1	1.57	0.165889
G06	Stat1	0.96	0.976210

G07	Tcf4	1.16	0.496318
**G08**	**Thbs2**	**2.18**	**0.015017**
G09	Tnni3	0.81	0.030529
G10	Tnnt2	0.79	0.007858
**G11**	**Ubb**	**0.48**	**0.004160**
G12	Zyx	0.98	0.799114
H01	Actb	1.09	0.334963
H02	B2m	0.82	0.196250
H03	Gapdh	0.63	0.007635
H04	Gusb	1.72	0.065314
H05	Hsp90ab1	1.04	0.471944

Supplemental Table S1: RT^2 Mouse Cardiovascular Disease Array PCR results. The fold change displayed is the fold change of the adenine-fed group against the control group's expression of each gene. This table contains all obtained by the Mouse Cardiovascular Disease array, with items **bolded** if their p-value is <0.05. For the Qiagen categorization of all the markers tested by the array, see https://geneglobe.qiagen.com/us/product-groups/rt2-profiler-pcr-arrays/PAMM-174Z
